# Nursing managers’ perceptions of teamwork and collaboration in mining primary healthcare clinics in Gauteng

**DOI:** 10.4102/curationis.v47i1.2574

**Published:** 2024-07-31

**Authors:** Sanele E. Nene

**Affiliations:** 1 Department of Nursing, Faculty of Health Sciences, University of Johannesburg, Johannesburg, South Africa

**Keywords:** perceptions, nurse managers, teamwork, collaboration, mining, primary healthcare

## Abstract

**Background:**

Teamwork and collaboration among nursing managers, nurses, doctors and peripheral hospitals treating mining patients is pivotal. A case study of specific mining primary healthcare clinics revealed a lack of teamwork among the doctors and nursing managers, even on decisions that show productivity.

**Objectives:**

The aim of this study was to explore and describe nursing managers’ perceptions of teamwork and collaboration in mining primary healthcare clinics in Gauteng.

**Method:**

A qualitative, exploratory, descriptive and contextual research design was adopted to conduct this study. Data were collected by conducting semi-structured individual interviews with 10 participants and thematically analysed. Data saturation was reached by the seventh participant and confirmed with three more interviews. Trustworthiness measures and ethical considerations were preserved as protocols because of the nature of the study.

**Results:**

Three themes emanated from the study: (1) team coordination and support improve teamwork and collaboration in primary healthcare clinics, (2) there is a lack of involvement from the nursing team, negatively influencing teamwork and collaboration and (3) collaboration can improve the quality of healthcare services rendered in mining primary healthcare clinics.

**Conclusion:**

All mining primary healthcare clinic team members should be involved in operational activities to foster teamwork and collaboration.

**Contribution:**

This study revealed that teamwork and collaboration should be facilitated to improve the quality of healthcare service in mining primary healthcare clinics.

## Introduction

In mining primary healthcare clinics, the practice of one nurse completing tasks is no longer an effective task-allocation method (Schot, Tummers & Noordegraaf 2020). Teamwork and collaboration among nurses, doctors and other stakeholders have instead been proven to be the most effective and efficient method of task allocation, decision-making and problem solving (Castañer & Oliveira 2020; Lemons & Strong 2016). The demands for primary healthcare services in the mining sector are increasing and influenced by challenges such as a lack of resources, slow economic growth, the rapid population increase and the high burden of diseases. Veldsman and Pauw (2018) and Cottong (2020) suggest that teamwork and collaboration can counteract these challenges.

Askerud and Conder (2017) and Nene, Ally and Nkosi (2020) define teamwork and collaboration in nursing as dynamic processes involving more than one nurse practitioner or healthcare professionals or disciplines exploring ideas and providing solutions to yield optimal healthcare services for healthcare users. Previous studies reported that nursing managers within healthcare establishments, whether in hospitals or primary healthcare settings, should remain cognisant that teamwork and collaboration do not merely refer to operational processes to complete tasks but should be considered as measurable key performance elements (Castañer & Oliveira 2020; Cottong 2020). Nurses should be able to commit and account for the tasks they completed through teamwork and collaboration in their performance management review meetings.

Alghamdi and Bach (2018) affirm that teamwork in nursing promotes the use of nurses’ skills to gain a competitive advantage and nursing managers should therefore consider teamwork as an essential facet of a successful organisation. Teamwork and collaboration enable nurses to execute tasks efficiently and effectively while creating an opportunity for the team to learn under the supervision of those who are more skilled. As stated by Hu and Liden (2015), Alghamdi and Bach (2018) and Nene (2022), teamwork and collaboration in primary healthcare should not be limited to nurses and doctors only, but disciplines outside healthcare, such as information communication and technology experts, building specialists and many others, should be allowed to express their expertise and promote the achievement of the healthcare organisation’s goals. However, Ryan (2017) and Nene et al. (2020) point out that effective teamwork and collaboration in nursing are difficult to achieve without open communication and adequate power distribution among nurses.

In their studies on teamwork and collaboration in primary healthcare settings, Alghamdi and Bach (2018) concluded that teamwork and collaboration lead to better productivity and greater efficiency as new ideas and creativity are generated through interdisciplinary integration. These authors also laud the fact that teamwork and collaboration can, among other things, keep nurses’ morale high as they feel the value of their involvement. A previous study revealed that teamwork and collaboration in nursing protect patients from poor nursing care, save time and prevent complications that may emerge as a result of patients waiting for care (Goh et al. 2020).

South Africa’s mining sector contributes a gross domestic product (GDP) of up to R351 billion per annum (Abuya 2018; Minerals Council South Africa 2020; Nene 2021). The mining company where this study was conducted is one of the country’s largest gold, uranium and platinum producers. It has onsite primary healthcare clinics and occupational health centres and employs healthcare workers to provide services to about 17 000 mine workers. In these primary healthcare clinics, 45 nurses report to eight unit managers. All the nurses and unit managers ultimately report to nursing managers. The nursing managers, unit managers and nursing staff work together in teams and collaborate with other healthcare professionals, such as doctors and physiotherapists, to provide comprehensive quality healthcare services to the mine workers.

### Problem statement

In the mining primary healthcare clinics where this study was conducted, the researcher has observed that nursing managers and doctors are not working as a team and are struggling to collaborate to ensure optimal patient care. They unnecessarily challenge each other’s decisions, and each party wants the final word. Hence, the researcher became interested in understanding nursing managers’ perceptions of teamwork and collaboration in these mining primary healthcare clinics.

According to Alghamdi and Bach (2018), to achieve and improve the operational outcomes in a primary healthcare facility, nurses and other healthcare professionals must work as a team and in collaboration, irrespective of their differences. Nurses and doctors should tackle the challenges and differences that prevent them from working as a collaborative team because primary healthcare is a multi-disciplinary approach (Nene 2022).

### Aim and objectives

This study aimed to explore and describe nursing managers’ perceptions of teamwork and collaboration in mining primary healthcare clinics in Gauteng.

## Research methods and design

### Research design

A qualitative, exploratory, descriptive and contextual research design was adopted to achieve the study’s objectives.

### Setting

This study was conducted in mining primary healthcare clinics located in Gauteng province in the West Rand of South Africa. The mining company built onsite primary healthcare clinics for its employees, and it employs nurses, unit managers, supporting staff and nursing managers. Four of these onsite clinics operate 24 h a day, and others are open for 8 h per day.

### Research methods

The following research methods were adopted in this study.

### Population and sampling strategy

The population for this study comprised all nursing managers working for a specific mining company. The sample of this study comprised 10 nursing managers who were selected from a total of 15 nursing managers using a non-probability purposive sampling strategy. Inclusion criteria for participation stipulated that nursing managers had to be employed at the mine’s primary healthcare clinics, with more than 1 year of work experience and be registered with the South African Nursing Council as professional or general nurses and nurse administrators. Nursing managers on leave during data collection and those working as occupational health nursing managers were excluded from this study as they were not exposed to primary healthcare clinics’ activities.

### Data collection

Semi-structured individual interviews were conducted from 01 September 2017 to 18 December 2017 to collect data from the nursing managers using English as a language of communication. Data were collected by the researcher, who worked in the mine as a case manager reporting to a group case manager. The group case manager reported to one of the nursing managers. The researcher did not have any personal relationship with any of the participants.

The participants were recruited through an email inviting them to attend an information session on the study, where those who were willing to participate signed informed consent forms. During the information session, participants asked questions about the study and data-collection process, and the researcher responded to those questions. Interviews were conducted in the participants’ offices on dates and at times convenient for their work schedule. Interviews lasted approximately 45–60 min, and field notes were documented during each interview to capture a true description of participants’ gestures and emotions (Creswell & Guetterman 2021). The central question posed to the participants was: ‘Is there teamwork and collaboration in this mining primary healthcare setting?’; follow-up questions were based on their responses and linked to the central question. Interactive communication strategies, such as clarifying, probing, paraphrasing and non-verbal communication techniques, like nodding and eye contact, were used to facilitate the interviews. Data saturation was reached by the seventh participant, and three more interviews were conducted to confirm data saturation.

### Data analysis

The researcher followed Giorgi’s method of thematic analysis to analyse the data, as presented in Table 1 (Holloway & Galvin 2017).

**TABLE 1:** Giorgi’s data-analysis steps.

Step	Description
Step 1	The researcher read and deeply engaged with the in-depth individual interview transcripts, audio recordings and field notes.
Step 2	Data were arranged into themes, main topics and sub-topics as they emanated using simple descriptive language.
Step 3	Data and the findings were shared with the independent coder, who also coded the data to confirm the researcher’s findings.
Step 4	A consensus meeting on the findings was held between the researcher and independent coder.

### Ethical considerations

The researcher upheld the following ethical principles throughout the study: autonomy and respect, fairness, beneficence and non-maleficence (Dhai 2019). Autonomy was upheld by obtaining permission to conduct the study from the University of Johannesburg Faculty of Health Sciences Research Ethics Committee (REC-01-73-2017) and the management of the mine where the study was conducted. The participants were informed about the study and decided whether to participate. Those who decided to participate were informed of their right to withdraw from the study at any time should they wish to do so. The principle of respect was ensured by treating all the participants with respect and addressing them using their titles, such as Mr, Mrs, etc. Fairness in this study was ensured by providing the participants with the same information about the study and equal time to participate. There were no direct benefits for the participants; however, the findings that could benefit the mining primary healthcare clinics were shared with them as promised by the researcher at the beginning of the study. The principle of non-maleficence was upheld as there was no harm envisaged or imposed on the participants during or after the study.

### Trustworthiness

Lincoln, Lynham and Guba’s (2011) trustworthiness strategies, which are also recommended by Creswell and Poth (2018), were adopted to ensure the findings’ trustworthiness. These strategies are credibility, dependability, confirmability and transferability. Credibility was ensured by collecting data using individual interviews and recording field notes during each interview. To ensure dependability, the researcher regularly engaged and had supervision sessions with the research supervisors throughout the study. The study’s confirmability was maintained through the independent coding of data and a consensus meeting on the findings of the study. Transferability was ensured by providing a dense description of the study’s context and the research methods adopted to conduct the study.

## Results

The participants’ demographics are presented next, followed by the findings of the study. The verbatim quotations from the participants are presented in black italics and field notes in bold. Table 2 reflects the demographics of the 10 participants who were interviewed until data saturation was reached. Their ages ranged between 38 and 60 years, four participants were males and six were females, nine were Africans and only one was a white person.

**TABLE 2:** Participant demographics.

Participants information	*n* (*N* = 10)	Years
**Gender**		
Men	4	-
Women	6	-
**Ethnicity**		
African	9	-
White	1	-
Age range (years)	-	38–60

Three themes emerged from the study, namely (1) Team coordination and support improve teamwork and collaboration in primary healthcare clinics. (2) There is a lack of involvement from the nursing team, negatively influencing teamwork and collaboration. (3) Collaboration can improve the quality of healthcare services rendered in mining primary healthcare clinics.

### Theme 1: Team coordination and support improve teamwork and collaboration in primary healthcare clinics

The participants revealed that team coordination and support promote teamwork and collaboration in their mining primary healthcare clinics. This finding is confirmed by the following quotations from the participants:

‘Look ensuring that our teams are coordinated and supported help us to promote teamwork and collaboration whether with doctors, department of health, mineral resources department, occupational health team etc.’ (Participant 3, 57 years, female)

‘We coordinate the teams and support them by facilitating operational activities and also to ensure that our team members are self-starters and self-driven, that is our priority to promote teamwork here.’ (Participant 1, 45 years, male)

‘What is also critical for us is making sure that the leaders of those smaller teams within our primary healthcare which includes nurses and doctors … know the process that needs to be follow[*ed*] and are also well supported.’ (Participant 5, 44 years, female)

‘Another thing is that divided people don’t move forward, we cannot afford to have teams that are pulling to opposite directions. Yes, we will have conflicts but as teams we have to put our differences aside, be coordinated, support one another, to ensure that we work together and in collaboration.’ (Participant 2, 60 years, female)

This theme illustrates that when teams are well coordinated and supported, they are able to work together and in collaboration, thus expediting optimal primary healthcare outcomes. Participants also reported that coordinated teams develop self-driven team members.

### Theme 2: There is a lack of involvement from the nursing team, negatively influencing teamwork and collaboration

The participants also mentioned a lack of nursing team involvement, negatively influencing teamwork and collaboration in the primary healthcare clinics. This view was affirmed by the following quotations:

‘To be honest with you at times we don’t involve our teams when we making decisions that are affecting them and this have to change otherwise we will never witness any teamwork or collaboration in our clinics.’ (Participant 8, 46 years, female)

‘I think for us managers, we lack saying to our nurses and doctors, make decisions, we will support you, can take us far as a team.’ (Participant 5, 44 years, female)

‘We really lack team involvement, whatever we need to implement to ensure promote teamwork, we have to distribute power to them so that they own their activities, and while doing that we also need to value them; respect them and you appreciate them.’ (Participant 7, 59 years, male)

‘We are not involving them enough, allowing team members to take turns on leading can assist us to achieve our major purpose, which is to support the mine to ensure that people are healthy and productive, that is the main major thing.’ (Participant 6, 59 years, male)

This theme reflected that a lack of nursing team involvement negatively influences teamwork in these mining primary healthcare clinics. The participants alluded that nursing managers have to distribute power among the nurses. They also added that nurses must take ownership of their activities and be appreciated by the nursing managers.

### Theme 3: Collaboration can improve the quality of healthcare services rendered in mining primary healthcare clinics

Another perception from the participants was that collaboration could improve the quality of healthcare services rendered in these mining primary healthcare clinics.

‘We have to ensure that we collaborate with relevant stakeholders such as [*the*] National Department of Mineral Resources one of as our main regulators, as this will ensure that we are following appropriate regulations thus improve the quality of our services.’ (Participant 4, 38 years, male)

‘Collaboration is the way, it should be Collaboration should be done in such a way that our patients receive quality healthcare services, hence it is important for us to know whom can we collaborate with and who can assist with what amongst them.’ (Participant 3, 57 years, female)

‘For collaboration to take place, one need to know that, we have in-house, what we call internal, stakeholders and external stakeholders, your human resource, mine workers, healthcare team and different hospitals we are referring our patients to.’ (Participant 5, 44 years, female)

‘Collaboration can take us to places, yes through it we can produce a very healthy workforce for the mines as it will result to improved quality healthcare service, however, let me tell you; we have to create a safe space for all those that we are collaborating with to ensure that the collaboration relationships are sustainable, you understand!.’ (Participant 10, 52 years, female)

The participants reported that collaboration can improve the quality of healthcare rendered in mining primary healthcare clinics. This is achieved by ensuring that relevant stakeholders collaborate, follow appropriate regulations and create a conducive environment for teamwork.

## Discussion

The study’s findings revealed that there is teamwork and collaboration in the mining primary healthcare clinics where the study was conducted, and it can improve the quality of services. However, the lack of nursing teams’ involvement in teamwork and collaboration should be addressed.

### Theme 1: Team coordination and support improve teamwork and collaboration in primary healthcare clinics

The participants shared that team coordination and support promote teamwork and collaboration in the primary healthcare clinics where they worked. A study by Ryan (2017) showed that organisations that ensure team coordination and support as an organisational culture have a good reputation and achieve their organisational objectives timeously. Alghamdi and Bach (2018) support this view by stating that well-coordinated and supported teams are the engineers of teamwork and collaboration, which is critical for the effective execution of operational activities to attain the organisation’s vision. Team coordination and support promote teamwork by creating self-starters and self-driven team members with a level of independence that allows for maximum performance, in collaboration and as a team (Ryan 2017). Al-Dossary (2017), Jooste (2017) and Nene (2021) revealed that strong leadership qualities such as strategic and critical thinking, enthusiasm, harmony and team empowerment are essential for team coordination and support. Mbebwo (2019) and Mofolo, Heunis and Kigozi (2019) also suggest that nursing managers should not only provide development and behavioural conduct support to the team but should also provide the resources required to execute tasks as this will enable team members to practice teamwork and collaboration in the clinics. It is clear that well-coordinated and supported teams reach excellent results. It would be difficult to ensure team coordination and support, which will assist the primary healthcare clinics in attaining their operational objectives timeously, if nursing managers do not possess strong leadership qualities and do not advocate for the resources required for operations in the clinics.

### Theme 2: There is a lack of involvement from the nursing team, negatively influencing teamwork and collaboration

The study’s findings revealed that the mining primary healthcare clinics lacked involvement from the nursing team, and this negatively influences teamwork and collaboration. Grenuk (2021) posits that a lack of nursing teams’ involvement and collaboration is caused by poor leadership, poor communication among team members, unclear roles and responsibilities, outdated technologies and a lack of empowerment. In support, Graystone (2019) and Nene (2021) claim, the absence of communication negatively affects teamwork and inhibits collaboration within organisations. According to McKnight and Moore (2022), nursing managers should address nursing teams’ involvement by employing shared governance in their leadership style as it allows for better teamwork and collaboration. The process of adopting shared governance can be challenging for nursing managers; it requires them to be open minded as it shifts the focus from a bureaucratic, top-down management style to a collaborative one from the administrative team to the nurses, doctors and other healthcare professionals providing care to the patients presenting at primary healthcare clinics (McKnight & Moore 2022; Nene et al. 2020). Crossler et al. (2017) emphasise that involving and allowing team members to lead their own activities improves their leadership skills and creativity. Alghamdi and Bach (2018) add that teams’ involvement builds trust between nursing managers and their team members. If nursing managers and their teams intend to achieve shared organisational goals, they have to manage the challenges that result in a lack of team involvement. This approach promotes team spirit, teamwork and collaboration, and it is no longer effective for team members to work independently as tasks are completed better and more effectively in teams.

### Theme 3: Collaboration can improve the quality of healthcare services rendered in mining primary healthcare clinics

The participants emphasised that collaboration improves the quality of healthcare services in these mining primary healthcare clinics. Castañer and Oliveira (2020) reveal that two is always better than one; therefore, it is essential for nursing managers to foster collaboration in mining primary healthcare clinics to improve the quality of services being rendered. Schot et al. (2020) outlined that collaboration can be achieved by ensuring adequate organisational arrangements such as clear, common rules and suitable information structures, as well as time, space and resources that enable professionals to get to know each other and work together. Jooste (2017) substantiated this view by positing stakeholders relevant for collaboration must be identified and factors that inhibit them from working together must be dealt with efficiently. Team members or health professionals have to decide whom they can collaborate with to execute a specific task and ensure that identified stakeholders for collaboration remain relevant to their obligations.

Schot et al. (2020) annotated that nursing managers, policy developers and all health professionals treating patients in mining primary healthcare clinics should conduct regular collaboration meetings to determine how their collaboration can improve the quality of healthcare being rendered. Collaboration saves time and costs, thus improving the quality of services. Consequently, Noordegraaf and Burns (2016) argue that nurses should break down the boundaries that separate them from developing collaborative models and collective decision-making with other professionals, such as statutory bodies, doctors and union members, and encourage their colleagues to participate.

Collaboration greatly benefits the sampled clinics; however, nursing managers must ensure that relevant stakeholders are identified for collaboration, and that working arrangements and rules are clearly stipulated by the parties involved (Mbebwo 2019). Regular stakeholder meetings must be held, and all parties should contribute equally during these engagements (Castañer & Oliveira 2020).

### Limitations

This study was qualitative and contextual in nature. Therefore, its findings are not generalisable to other mining primary healthcare clinics that did not form part of the sample of this study. Some participants were scared to admit their negative attitudes towards their leadership roles even though they were informed that their participation in the study was anonymous and confidential.

### Recommendations

The following recommendations are made for nursing practice and policy, nursing research and education.

### Nursing practice and policy

It is recommended that the operational activities in mining primary healthcare clinics should foster teamwork and collaboration to improve the quality of services being rendered to patients. A policy on teamwork and collaboration should be developed for these clinics.

### Nursing research

Research on teamwork and collaboration should be extended to primary healthcare medical doctors, nurses and other healthcare workers providing services to mining patients. A qualitative study should be conducted to evaluate and verify whether this study’s findings are still the same or have changed as it was conducted in 2017, and an evaluation of the quality of teamwork and collaboration in primary healthcare clinics is recommended.

### Nursing education

The findings of this study should be integrated into the curriculum of post-graduate diplomas in nursing to ensure that current and future nursing specialists clearly understand the potential benefits and effects of teamwork and collaboration.

## Conclusion

Despite the challenge of a lack of team involvement, which should be addressed, there is teamwork and collaboration in the mining primary healthcare clinics, which should be encouraged to improve the quality of services being rendered.
